# Treatment of Severe Hypertriglyceridemia with Continuous Insulin Infusion

**DOI:** 10.1155/2011/293917

**Published:** 2011-06-27

**Authors:** Yesica Rodríguez Santana, Andrea Nimo Román, Iker García Sáez, José Manuel López Alvarez, Eduardo Consuegra Llapur, Rafael González Jorge

**Affiliations:** Pediatric Intensive Care Unit, Hospital Universitario Materno-Infantil, 35016 Las Palmas de Gran Canaria, Spain

## Abstract

Severe hypertriglyceridemia (SH) represents a therapeutic emergency because of the possibility of developing cardiovascular events and hyperlipemic acute pancreatitis (PA). Most patients with SH suffer primary or genetic abnormality in lipid metabolism in combination with a precipitating factor such as uncontrolled diabetes mellitus, alcoholism, and drug intake. The standard treatment of hypertriglyceridemia (HTG) with omega 3 fatty acids and fibrates, along with dietary changes, has no effect on an emergency situation. There are no clinical guidelines to SH, but therapy with insulin, heparin, a combination of both, plasmapheresis, or octreotide have been tested succesfully. We report the case of a 10-year-old girl with clinical acute pancreatitis and diabetic ketoacidosis debut, along with incidental finding of an SH, who had a good outcome after treatment with insulin intravenous infusion.

## 1. Case Report

A ten-year-old girl previously healthy, except allergic rhinitis caused by mites at the age of three was admitted to Intensive Care Unit (ICU) because of abdominal pain, anorexia, polydipsia, vomiting, and hyperventilation. She was diagnosed with acute pancreatitis and diabetic ketoacidosis [1, 2].

On physical examination, she presents with (a) BP 115/74 mm Hg, HR 128 bpm, RR 23 rpm, Temp 37°C, O2 Sat 98%, (b) decay and signs of moderate-severe dehydration (pale skin, sunken eyes, oral mouth typically dry, and Kussmaul breathing), (c) normal cardiopulmonary auscultation, and (d) spontaneous abdominal pain that intensifies with palpation and hepatomegaly of 3 cm below costal margin.

Lab tests show a severe metabolic acidosis (venous gasometry: pH 6.76, pCO2 7.3 mm Hg, HCO3 1.3 mmol/L, BE −31 mmol/L), glucose 472 mg/dL, amylase 700 U/L, alkaline phosphatase 100 U/L, and LDH 280 U/L. In Chest-abdomen X-ray, a gastric dilatation is evident, with no other findings. ECG: incomplete right bundle branch block without signs of myocardial ischemia. Abdominal ultrasound shows signs of liver disease and/or fatty liver. Abdominal CT: moderate amount of free abdominal fluid. No objective findings of liver or pancreatic involvement were observed.

Acute pancreatitis was treated with fasting, gastric decompression by nasogastric tube, and analgesia with meperidine and IV magnesic metamizole. Fluid therapy was also initiated and intravenous bicarbonate. In addition, we started insulin continuous perfusion for diabetic ketoacidosis treatment [2–5].

**Figure 1:**
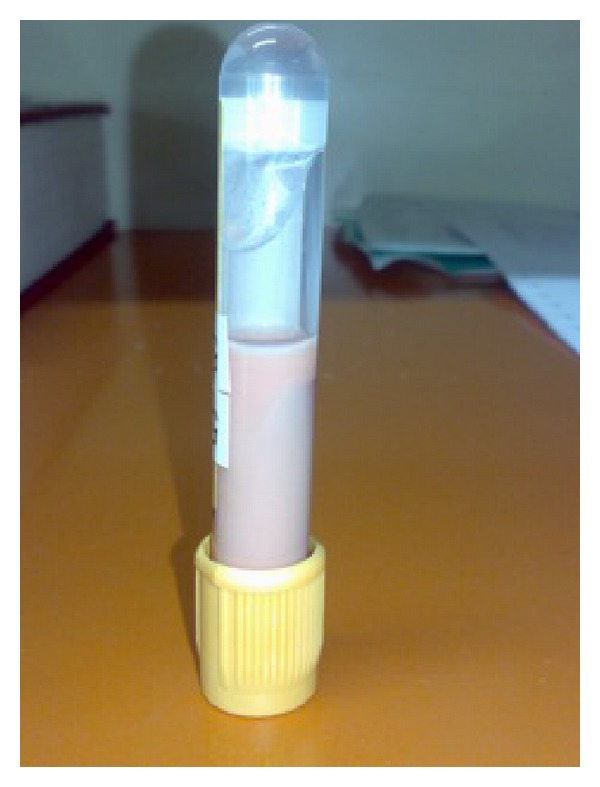
Patient's hyperlipemic serum.

Due to the lipemic appearance of serum (Figure 1), we performed a lipid profile, showing high levels of triglycerides (TGC) 10,260 mg/dL and total cholesterol 970 mg/dL (non-HDL 924 mg/dL). By the finding of an SH, we consider emergency therapeutic options (plasmapheresis, intravenous infusion of heparin, and/or insulin) and we chose the latter.

**Figure 2:**
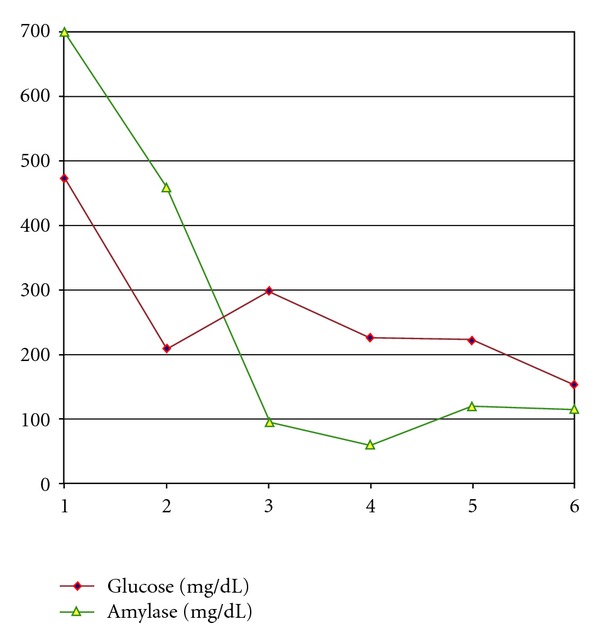
Daily evolution of glucose and amylase levels during the first six hospitalization days.

**Figure 3:**
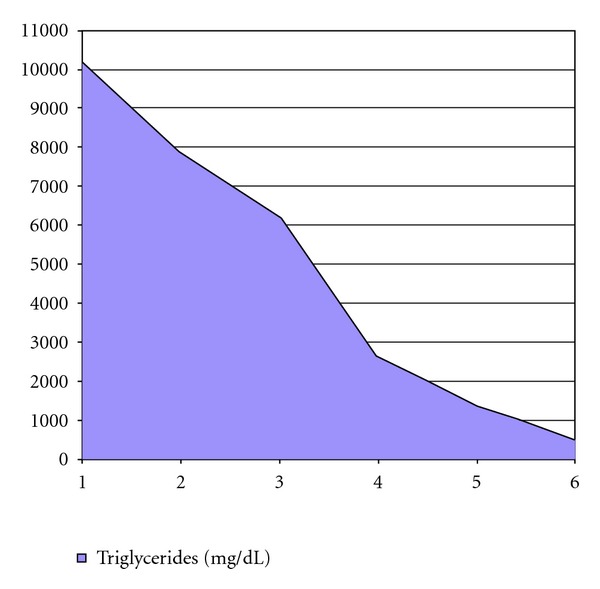
Serum triglycerides levels during the first six hospitalization days.

During the first 24 hours, vascular expansion and dopamine support (10 mcg/kg/h) due to sustained hypotension was required. A progressive normalization of blood glucose, acidosis, and ketonuria was achieved. Triglyceride levels decreased progressively so insulin infusion remained at 0.5 to 1 IU/kg/h and supply of glucose controlled. At 48 hours, TGC levels dropped to 6174 mg/dL, decreasing to 476 mg/dLs at 96 hours (Figures 2 and 3). During her stay in ICU, there were no neurological disorders, respiratory or kidney problems, and she did not experience abdominal pain after discontinuation of analgesia within 24 hours after admission. There were no bleeding and no clinical or laboratory signs of infection. About 48 hours after admission, we started lipid-free parenteral nutrition. She was discharged from the ICU after 72 hours, following clinical stabilization in pediatric ward where she remained hospitalized 2 weeks.

After two months of monitoring, clinical evolution was found favorable. Asymptomatic, TGC level was 82 mg/dL, total cholesterol 173 mg/dL (HDL 41 mg/dL and LDL 116 mg/dL), amylase 61 U/L, lipase 12 U/L (both normal). ALT, AST, and GGT were also normal. Lipoprotein determinations were A1: 25 mg/dL (115–210 mg/dl), B: 62 mg/dL (55–135 mg/dL) B/A1 Ratio: 2.48 mg/dL, E: 614 mg/dL (0–94), CII: 239 mg/dL (15–40). Insulin antibodies were negative, antiGAD 21.5 mg/Dl U/mL (0-1), and anti-tyrosine phosphatasa 2.72 U/mL. Glycemia were well controlled with subcutaneous insulin regimen (23 U Lantus and Humalog 4-3-5 U), with HbA1c 7.9%, and negative glucosuria and ketonuria.

## 2. Pathophysiology and Discussion

Hypertriglyceridemia may cause AP in up to 7% of cases. This is the most common cause of acute biliary AP after alcohol [1, 6, 7]; however, it rarely occurs except when triglyceride levels exceed 1500 mg/dL [6, 7]. Nevertheless, the detection of mild to moderately elevated levels of triglycerides (200–1000 mg/dL) usually occurs in the early stages of the AP of any etiology; that is, the HTG can develop in the context of endocrine dysfunction that accompanies the AP, or it may be the precipitating cause of it [6].

On the other hand, such high levels of triglycerides may indicate some sort of defect in lipid metabolism, a hypothesis that has been confirmed by several works that have found abnormalities or deficiencies of key enzymes in triglyceride metabolism, as example, mutations in the gene for lipoprotein lipase enzyme (LPL) located on chromosome 8 [1, 6, 8].

The enzyme LPL is expressed in capillary endothelium of muscle and adipose tissue and is responsible for hydrolyzing triglycerides, releasing fatty acids [2], which will be captured and “internalized” by the cells. An altered or decreased activity of LPL may lead to an increase in circulating triglycerides. On the other hand, it has been observed that to reach levels high enough to develop clinically relevant complications, a condition that also affects the general metabolism [1] and acts as a trigger (diabetes mellitus, alcoholism, pregnancy, hypothyroidism, or certain drugs such as estrogens, furosemide, isotretinoin, tamoxifen, and *β*-blockers) has to coexist [2]. It is estimated that 75% of patients with pancreatitis due to hypertriglyceridemia have alcoholism or diabetes mellitus poorly controlled [9, 10].

Isolated cases of PA due to hypertriglyceridemia have been reported and treated with plasma or lipoprotein apheresis with favorable response [2]. However, the recognition of a decreased activity of the LPL enzyme as a key factor in the development of SH [2, 9, 10] has enabled the development of new therapeutic options, such as intravenous administration of insulin or heparin [1]. Both activate the LPL bound to endothelium. In addition, heparin mobilizes and releases the enzyme of the endothelium to plasma [1, 2]. Insulin promotes the synthesis of LPL and stimulates the uptake of fatty acids released from triglyceride hydrolysis by LPL itself [1, 2]. 

Octreotide has also been used, a somatostatin analogue, because it uses its receptors in the pancreas, modulating pancreatic exocrine secretion [2].

In the case presented, triglyceride levels reached 10260 mg/dL, with an amylase also high, and clinical manifestations compatible with AP, in the context of diabetic ketoacidosis. When initiating treatment with insulin we not only got a glycemic control, but there was also a dramatic reduction in the HTG to values considered safe (triglycerides <500 mg/dL) after 96 hours of admission. Therefore, after normalization of blood glucose and correction of ketosis, it was decided to continue with a continuous infusion of insulin from 0.5 to 1 IU/kg/h, achieving standardization practice triglyceride levels. While experience in pediatric patients is very limited and the presentation through SH is unusual, we believe that treatment with intravenous insulin is an alternative that is fast and secure.
